# Quantitative evaluation of severity of behavioral and psychological symptoms of dementia in patients with vascular dementia

**DOI:** 10.1186/2047-9158-2-9

**Published:** 2013-04-22

**Authors:** Wei-Dong Pan, Sohei Yoshida, Qian Liu, Chun-Lan Wu, Jun Wang, Jin Zhu, Ding-Fang Cai

**Affiliations:** 1 Department of Neurology, Shuguang Hospital Affiliated to Shanghai University of TCM, 185, Pu-An Road, Shanghai, 200021, China; 2 Department of Neurology, Kansai University Clinic, Kansai University of Health Sciences, 1-11-2 Wakaba, Kumatori-Cho, Sennan-Gun, Osaka, 590-0482, Japan; 3 Department of Cardiology, Shuguang Hospital Affiliated to Shanghai University of TCM, 185, Pu-An Road, Shanghai, 200021, China; 4 Laboratory of Neurology, Institute of Integrative Medicine, Zhongshan Hospital, Fudan University, 180, Fenglin Road, Shanghai, 200032, China

**Keywords:** Vascular dementia, Behavioral and psychological symptoms of dementia, Rating scale of the behavioral pathology in Alzheimer’s disease, Neuropsychiatric inventory, Actigraph

## Abstract

To quantitatively evaluate severity of behavioral and psychological symptoms of dementia (BPSD) for vascular dementia (VD). Changes of 51 patients with VD in BPSD between the first and 24th week were assessed using the Neuropsychiatric Inventory (NPI) and the behavioral pathology in Alzheimer’s disease (BEHAVE-AD) rating scale, in detrended fluctuation analysis (DFA) represented by diurnal activity (DA), evening activity (EA), and nocturnal activity (NA), and the relationships were analyzed. The subscores of activity disturbances, diurnal rhythm disturbances, and anxieties and phobias in the BEHAVE-AD score, and that of agitation, irritability, and sleep disorder in the NPI score were significantly increased compared with the first week, as was for the changes for EA in the DFA value. A linear correlation was observed between the changes of activity disturbances plus anxieties and phobias, and those of DA, and between the development of diurnal rhythm and those of EA, the vehement and autism scores and those of DA, and the difference in sleep disorder scores and those of EA, respectively. Analysis of DA, NA, and EA may reflect the fluctuational degrees of VD-BPSD, can provide a useful assessment of VD-BPSD accompanied by clinical scores for VD.

## Introduction

With aging populations, the prevalence of dementia will continue to rise in the coming decades [1,2]. Vascular dementia (VD) is associated with the presence of atherosclerosis and this association applies to subjects clinically diagnosed with vascular-type dementia as well as those with Alzheimer’s disease (AD) [3]. The behavioral and psychological symptoms of dementia (BPSD), such as agitation, aggressive behavior, day-night rhythm disturbances, mood alterations, and hallucinations are among the most prominent clinical features seen during the late course of dementia such as AD or VD. Of all these symptoms, agitation places a particular burden on professional caregivers and family members during home care [3,4]. The behavioral pathology in Alzheimer’s disease (BEHAVE-AD) rating scale [5] is frequently used to monitor BPSD, however, these BEHAVE-AD assessments are subjective and place great demands on the personnel resources of the hospital. The Neuropsychiatric Inventory (NPI) [6] is scored through a semi-structured interview by a clinician or researcher with a caregiver of the person with dementia, and it can be administered and scored as a 10-item (excluding sleep and vegetative symptoms) or a 12-item instrument (including both). The clinical scores may not adequately reflect the fluctuations of physical activities and disease severity [7]. Recently, by using a method to analyze physical activity recorded by actigraph, Pan, et al. [7-9] have quantitatively evaluated the severity of motor fluctuation and sleep disorders in patients with Parkinsonism. Previous studies examined the psychiatric symptoms associated with actigraph in subjects with dementia or other psychiatric disorders [10-12]. Wrist actigraph is a rater-independent method for obtaining data on motor activity and has been shown to be a valid means of measuring agitation and sleep-wake rhythms in patients order patients [13-15]. Moreover, it is a particularly attractive instrument in clinical care, as it causes minimal distress to the patient. Thus, the aims of the present study were to assess the probability of changes in analytical parameters, such as the detrended fluctuation analysis (DFA) values of diurnal activity (DA), evening activity (EA), and nocturnal activity (NA) using this quantitative device, and to compare these values with the clinical scores of BEHAVE-AD and the NPI to obtain a pilot, objective scale representing severity of VD-BPSD.

## Methods

### Subject inclusion

Subjects with Mini-Mental State Examination (MMSE) scores between 10–24 and satisfying the fourth edition of the Diagnostic and Statistical Manual of Mental Disorders DSM-IV-TR Fourth Edition (DSM-IV) for dementia from June 2009 to December 2011 at the Department of Neurology of Shuguang Hospital Affiliated to Shanghai University of TCM were recruited into the study. We examined 138 patients who had been diagnosed with AD, mild cognitive impairment, frontal-temporal dementia, Parkinsonism-dementia, or Lewy body dementia. After excluding 12 patients suffering primarily from brainstem infarction, whose inferior activity would influence the evaluation, only 56 patients who suffered from VD (mean age ± SD, 60.2 ± 9.7 years old, mean duration of illness, 6.9 ± 5.2 years) were found to be suitable for this research. The diagnosis was multi-infarct dementia in 26 and subcortical vascular dementia in 30: these 56 study subjects satisfied the criteria for probable VD in accordance with the NINDS-AIREN criteria [16]. We did not include patients with white matter lesions, caused by specific etiologies, such as multiple sclerosis, brain irradiation, collagen vascular disease, and genetic forms of vascular dementia (such as CADASIL or CARASIL), or patients who showed signs of normal pressure hydrocephalus, previous brain tumors, or a previous diagnosis of major stroke or brain haemorrhage. Patients with a previous major psychiatric illness such as schizophrenia, bipolar disorder, psychosis, or compulsive-obsessive disorder, a central nervous system disorder, or alcoholism were also excluded. The study was approved by The Ethics Committee of Shuguang Hospital Affiliated to Shanghai University of TCM, and was performed under the principles outlined in the Declaration of Helsinki.

### Drug administration

Anti-dementia drugs were administered as described in Table 1. Patients were instructed to add or change anti-dementia drugs for at least 2 weeks (mean ± SD, 15.6 ± 7.5), and they were then evaluated again. They were not treated by any complementary and/or alternative treatments such as traditional Chinese medicine, Tai Chi exercise, or acupuncture.

**Table 1:** **Background characteristics of the patients with vascular dementia (VD) (**$\bar{X}\pm s$**)**

**Characteristic**	**Number**	**1st week**	**24th week**
Age (years)		60.2 ± 9.7	
Sex (M/F)		39/17	
Duration of VD (years)		6.9 ± 5.2	
MMSE		12.1 ± 1.7	10.9 ± 1.6
Huperzine A (μg/d)	n = 24	323.30 ± 173.9	358.30 ± 191.4
Aniracetam (mg/d)	n = 26	489.6 ± 179.3	506.6 ± 108.7
Memantine Hydrochloride (mg/d)	n = 30	6.67 ± 5.38	6.98 ± 4.47
Donepezil Hydrochloride (mg/d)	n = 16	8.21 ± 3.76	9.33 ± 6.94
Rivastigmine (mg/d)	n = 13	3.33 ± 1.66	3.75 ± 1.66
Galantamine Reminyl (mg/d)	n = 16	25.65 ± 22.36	26.82 ± 22.91

MMSE indicates Mini–mental state examination. Cognitive function drug administration: 1 class of drug 73.2%; 2 classes 23.6%; 3 classes 3.2%.

### Assessments

BEHAVE-AD [5]: BEHAVE-AD is the most widely used instrument for the evaluation of dementia-related behavioral changes. It addresses delusions, hallucinations, activity disturbances, aggressiveness, diurnal rhythm disturbances, affective disturbances, and anxieties and phobias. The BEHAVE-AD scores of all patients were evaluated on the day before the actigraph recordings in the series time windows (6 weeks each) during the 24-week follow-up by the same neurologists.

The Neuropsychiatric Inventory (NPI) [6]: NPI was used to assess 10 behavioral disturbances occurring in patients: delusions, hallucinations, dysphoria, anxiety, agitation/aggression, euphoria, disinhibition, irritability/ability, apathy, and aberrant motor activity. The NPI scores were assessed based on information from the patients or caregivers using the same time windows as when evaluating BEHAVE-AD.

Analysis of actigraphy: All patients wore a small watch-type activity monitor equipped with a computer (*MicroMini-Motionlogger*, Ambulatory Monitoring, Inc, Ardsley, New York) on the wrist of their non-dominant hand for 7 consecutive days in the series time windows (6 weeks each) during the 24-week follow-up. Data acquired during the diurnal activity (DA, between 6 a.m. and 6 p.m.), evening activity (EA, between 6 p.m. and 9 p.m.) and nocturnal activity (NA, between 9 a.m. and 6 a.m.) periods were used in the analyses. Discontinuous data were combined using an integrative method [7], and then analyzed by detrended fluctuation analysis (DFA), which evaluates the correlations between time scales and magnitudes of fluctuation (standard deviations) within each time scale [17,18]. More correlated signals represent a greater growth of the fluctuation magnitude with increasing time scale or length of data windows [7].

### Statistical analysis

The changes in BEHAVE-AD scores (including subscores), NPI scores (including subscores) and each DFA value (α) following the series time windows (for each 6 weeks) were compared with baseline (first week) using the Wilcoxon signed rank test. Pearson’s bivariate correlations were used to test the correlation between the changes in the BEHAVE scores and the DFA values, NPI scores, and DFA values, respectively. A significant difference was defined as *P* <0.05. SPSS Windows Version 17.0 was used for statistical analyses. All data are expressed as the mean ± standard deviation.

## Results

Five patients with VD (4 males, 1 female) dropped out of the study: 4 patients reported being inconvenienced by the actigraph (n = 3) or a negative effect on sleep (n = 1) when wearing the actigraph on their non-dominant wrists and refused to continue the research; the remaining patient destroyed the equipment twice and refused to wear it, and thus was withdrawn from the study, 51 patients completed this study.

**Figure 1:**
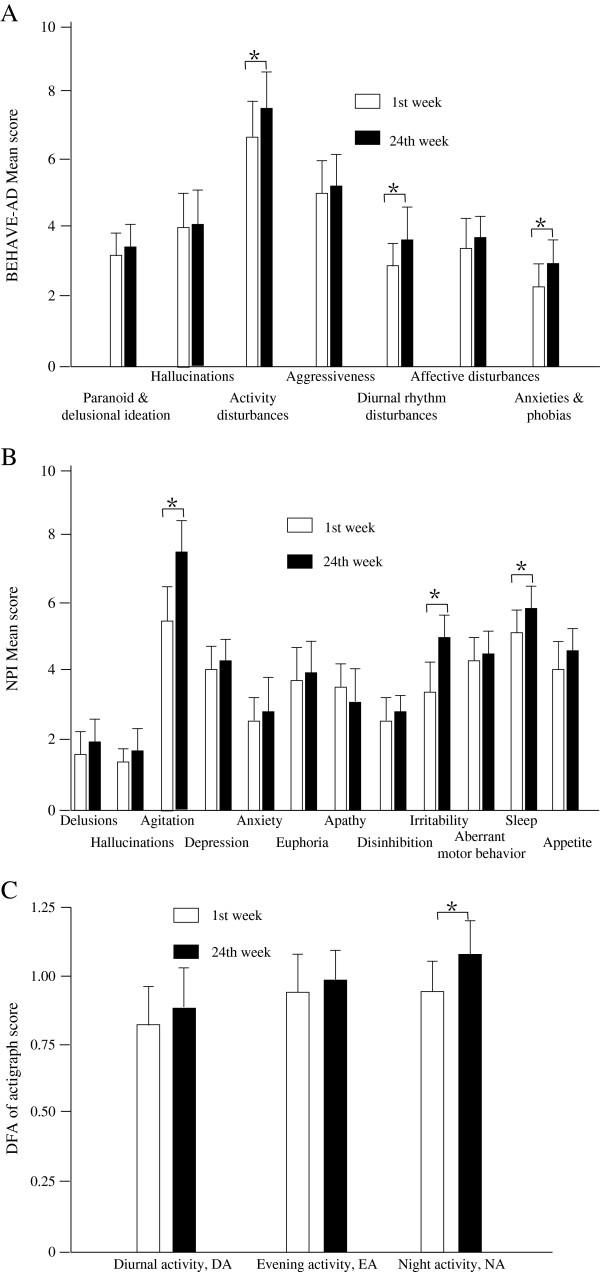
**The changes of behavioral and psychological symptoms and physical activity parameters of VD patients for patients with vascular dementia.** Changes in each subscore for BEHAVE-AD (**A**) and NPI (**B**), and changes in diurnal activity (DA), evening activity (EA), and nocturnal activity (NA) (**C**) in the analysis of actigraph recordings between first week and 24th week in patients with vascular dementia. * p < 0.05, compared with first week. BEHAVE-AD: rating scale of the behavioral pathology in Alzheimer’s disease; NPI: Neuropsychiatric Inventory.

By the end of the 24-week follow-up period, all patients with VD appeared to exhibit increased BEHAVE-AD total and NPI total scores compared with baseline, although no statistically significant differences were observed (p = 0.82 and 0.79, respectively). Significant and persistent increases compared with baseline were found in the BEHAVE-AD subscores of activity disturbances, diurnal rhythm disturbances, and anxieties and phobias, and in the NPI subscores of agitation, ignitability, and sleep disturbance (Figure 1A, B and Table 2). The α of the DFA values of NA but not DA and EA had increased significantly by the end of 24 weeks compared with baseline (p = 0.037, 0.051 and 0.052, Figure 1C and Table 2).

**Table 2:** **Behavioral and psychological symptoms and physical activity parameters of VD patients (*x*** **±** ***s*)**

**Parameter**	**Time**	
	**1st week**	**24th week**
**BEHAVE-AD**		
Paranoid and delusion ideation	3.05 ± 0.38	3.26 ± 0.36
Hallucinations	3.82 ± 0.52	3.95 ± 0.61
Activity disturbances	6.55 ± 0.68	7.34 ± 0.71*
Aggressiveness	5.06 ± 0.30	5.12 ± 0.29
Diurnal rhythm disturbances	2.81 ± 0.33	3.45 ± 0.42*
Affective disturbances	3.05 ± 0.43	3.28 ± 0.35
Anxieties and phobias	2.27 ± 0.35	3.19 ± 0.38*
**NPI Mean score**		
Delusions	1.55 ± 0.69	1.83 ± 0.56
Hallucinations	1.30 ± 0.51	1.58 ± 0.47
Agitation	5.55 ± 0.90	7.48 ± 0.88*
Depression	4.21 ± 0.81	4.29 ± 0.36
Anxiety	2.32 ± 0.81	2.44 ± 0.91
Euphoria	3.75 ± 0.65	3.82 ± 0.76
Apathy	3.33 ± 0.71	3.09 ± 0.80
Disinhibition	2.75 ± 0.68	2.91 ± 0.77
Ignitability	3.25 ± 0.92	5.17 ± 0.83*
Aberrant motor behavior	4.25 ± 0.69	4.38 ± 0.66
Sleep disturbance	5.32 ± 0.83	6.05 ± 0.69*
Appetite	4.08 ± 0.57	4.38 ± 0.61
**DFA of actigraph activity**		
Diurnal activity	0.82 ± 0.17	0.86 ± 0.14
Evening activity	0.87 ± 0.13	0.90 ± 0.11
Nocturnal activity	0.93 ± 0.16	1.03 ± 0.16*

Data presented are mean ± SD, * p < 0.05, compared with first week by Wilcoxon signed rank test.

**Figure 2:**
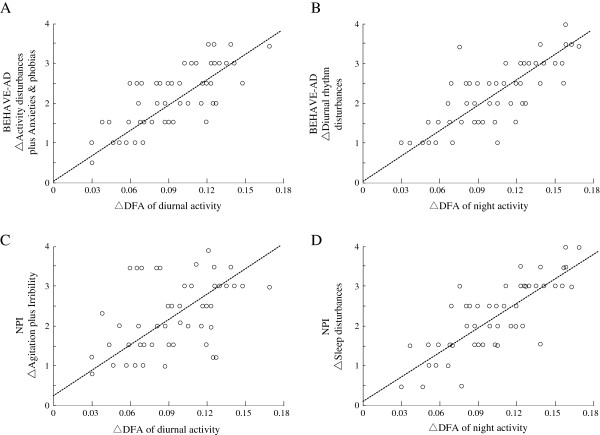
**The relationship of the changes between behavioral and psychological symptoms and physical activity parameters of VD patients for patients with vascular dementia.** Relationship of the changes in activity disturbances plus anxieties and phobias and diurnal activity (**A**), diurnal rhythm and evening activity (**B**) of the subscores for BEHAVE-AD, and relationship of the changes in agitation plus irritability and diurnal activity (**C**), and sleep disturbances and evening activity (**D**), respectively. (*r* = 0.674, 0.721, 0.668, and 0.809, *P* < 0.05, by Spearman’s rank correlation coefficient).

Low correlation coefficients were found between the changes in total BEHAVE-AD score and changes in DA, EA and NA of α values (r = 0.438, 0.367 and 0.479; p = 0.816, 0.521 and 0.673), and the changes in NPI total scores and the changes in DA, EA and NA of α values (r = 0.389, 0.472 and 0.318; p = 0.82, 0.809 and 0.67). A linear correlation coefficient of 0.674 (p = 0.03) between the changes in activity disturbances score plus anxieties and phobias scores of BEHAVE-AD and the changes in DA of α values, and a linear correlation coefficient of 0.721 (p = 0.042) between the changes in diurnal rhythm disturbance subscores of BEHAVE-AD and the changes in NA of α values were observed. Linear correlation coefficients were also observed between the changes in agitation plus irritability subscores of the NPI score and the changes in DA of α values (r = 0.668, p = 0.043, Figure 2A and B), and the changes in sleep disturbances subscore and the changes in NA of α values (r = 0.809, p = 0.022, Figure 2C and D).

## Discussion

The severities of some neurological and psychological diseases can now be quantitatively and objectively evaluated using modified analysis of actigraphic recordings[12-15,17-21]. Actigraphic methods might be highly specific and highly sensitive methods for analyzing movement disorders and non-motor disturbances associated with Parkinson’s disease [22]. Actigraphs are useful for evaluating the severity of various neurological diseases by analytical quantitative methods, and even for evaluating the effects of medications [23].

Honma et al. [24] evaluated the severity of biorhythm fluctuations in demented patients with delirium using law activity patterns, and attempted to develop a method that can help predict a prognosis or therapeutic decisions for such patients. Rochelle et al. [25] compared the effects of melatonin on sleep disorders using actigraphic recordings, Sleep Disorders Inventory (SDI), and sleep behavior and sleep quality ratings (SQR). They found the actigraphic sleep patterns showed a linear correlation with melatonin, although the correlation was lower than the SDI scores and sleep behaviors and SQR, and suggested the lower sensitivity of the actigraphic assessment might be caused by the limited number of subjects and the shorter study period.

Because most healthy controls exhibited no changes in activities or DFA scores in the 54 w records, while age-matched patients with PD demonstrated significant changes compared with before (Weidong P, unpublished observation, Tokyo University, Japan), we did not use a control group for comparison in this study. We studied VD patients for 24 weeks with modifying their treatments or doses of dementia medications. The BEHAVE-AD total scores and NPI total scores at the end of the study (after 24 weeks) were only slightly different to those at baseline, although some subscores, such as sleep patterns, had changed markedly compared with their baselines (Figure 1 and Table 2). Not only the sleep patterns of BEHAVE-AD and NPI but also the activity patterns of DFA from actigraph recordings demonstrated fluctuations at the end of 24 weeks compared with the baseline, and the correlations between them were high (Figure 2B and C). The results showed that the DFA patterns from actigraph may be useful for evaluating the severity of sleep disturbance, which is one symptom included in VD-BPSD, because values similar to those of clinical patterns were observed. Some of the degrees of change in clinical patterns of BEHAVE-AD and NPI were the same as the degrees of change in DFA patterns (Figure 2A and C). Evaluation of the changes in DFA values may represent a more quantitative and accurate method for assessing the disease severity or effects of dementia medications in conjunction with the clinical patterns.

Although the present findings revealed similar trends for DFA values from actigraph and the clinical patterns for VD-BPSD, there were no significant differences in some patterns because of the limited number of patients and the short study period (24 weeks). Moreover, although DFA parameters partially reflect the severity of VD-BPSD, it is essentially not evaluated using body movements. If a patient has movement disorders, such as tremors, the patterns might be markedly influenced [7]. The DFA method showed rough function to remove same frequency activities, but it is still unsatisfactory and a special assessment method may be needed to provide a more accurate method for the quantitative evaluation of VD-BPSD. On the other hand, the size of the actigraph may be bothersome to subjects who are very small, however, few dementia patients have repeatedly destroyed the equipment. The dependency of research and safety of the patient wearing an actigraph should be further studied and discussed.

In conclusion, the present preliminary data have replicated the previous finding that DFA, which determines the deviations in 3 parameters (DA, EA and NA) obtained by actigraph recordings, can be quantitatively used for assessing the severity of VD-BPSD in patients suffering from VD. The small sample size and single-blinded design are limitations of our pilot study. In addition, normative data for both healthy elderly and BPSD patients need to be established. Actigraph may be feasible and useful when predicting a prognosis or making therapeutic decisions for patients with VD-BPSD.

## Abbreviations

VD: Vascular dementia; BPSD: Behavioral and psychological symptoms of dementia; NPI: Neuropsychiatric Inventory; BEHAVE-AD: Behavioral pathology in Alzheimer’s disease; DFA: Detrended fluctuation analysis.

## Competing interests

The authors declare that they have no competing interests.

## Authors’ contributions

W-DP, participated in the entire study, formulated the study concept and design, provided statistical expertise and assisted with drafting of the manuscript; SY, participated in the entire study, assisted with concept and design, and drafting of the manuscript; QL, participated part of content, data compilation; C-LW, participated part of content, data compilation. JW, participated part of content, critical reversion of the manuscript for important intellectual content. JZ, participated part of content, data compilation. D-FC, participated in the part study, formulated the study concept and design, provided statistical expertise and assisted with drafting of the manuscript. All authors read and approved the final manuscript.
